# No-Risk, At-Risk, and High-Risk Middle School and High School Students: Contributions of the Quadripartite Model for Psychological Distress Prevention Programs

**DOI:** 10.3390/children12091188

**Published:** 2025-09-05

**Authors:** Marina Carvalho, Cátia Branquinho, Catarina Noronha, Nuno Neto Rodrigues, Tânia Gaspar, Margarida Gaspar de Matos

**Affiliations:** 1 Equipa Aventura Social, 1675-184 Lisboa, Portugal; marina.carvalho@ismat.pt (M.C.); cbranquinho@ucp.pt (C.B.); catarinanoronha26@live.com.pt (C.N.); tania.gaspar@ulusofona.pt (T.G.); 2 Instituto de Saúde Ambiental, Universidade de Lisboa, 1649-026 Lisboa, Portugal; 3 Faculdade de Ciências Humanas (FCH), Universidade Católica Portuguesa (UCPF), 1649-023 Lisboa, Portugal; 4 Direção-Geral de Estatísticas da Educação e Ciência, 1399-054 Lisbon, Portugal; nuno.rodrigues@dgeec.medu.pt; 5 School of Psychology and Life Sciences, Universidade Lusófona de Humanidades e Technologias (ULHT), 1749-024 Lisbon, Portugal; 6 The Applied Psychology Research Center Capabilities & Inclusion (APPsyCI), School of Psychology and Behavioral Sciences, Instituto Universitário (ISPA), Rua Jardim do Tabaco 34, 1149-041 Lisboa, Portugal

**Keywords:** quadripartite model, well-being, psychological symptoms, socio-emotional skills, students

## Abstract

**Highlights:**

**What are the main findings?**

- The quadripartite model is a useful tool for assessing psychological health in young people, considering psychological symptoms along with well-being.
- Young people with complete psychological health were distinguished from young people with complete psychological distress by having more socio-emotional skills.

**What is the implication of the main finding?**

- Prevention programs directed to the promotion of psychological health should consider the promotion of socio-emotional skills along with the modification of psychological symptoms.
- Public policies involving young people’s health and education would benefit from including both targeted and indicated school and community prevention programs.

**Abstract:**

Background/Objectives: Students’ psychological health problems have been widely studied for a long time. However, with the COVID-19 pandemic and due to the additional challenges related to the need for individual and contextual adjustment, a more comprehensive approach to psychological health and well-being is needed. The main goal of the present study was to identify the individual and contextual factors that could discriminate middle school and high school students based on well-being and psychological symptoms. Methods: In this study, carried out within the scope of the second wave of the study “Psychological Health and Wellbeing | School Observatory”, promoted by the Ministry of Education, 3037 students from different regions and levels of public education in Portugal, 49.5% female, aged between 9 and 18 years, participated by completing a research protocol after informed consent was given. Results: Cluster analysis allowed the identification of four groups based on the scores of well-being and psychological symptoms: complete psychological health, incomplete psychological distress, incomplete psychological health and complete psychological distress. The analysis of discriminant variables additionally showed relevant differences between the two extreme groups: complete psychological health students reported higher socio-emotional skills, whereas complete psychological distress students reported higher stress and anxiety scores and low life satisfaction. Conclusions: The obtained results highlight the need for early identification of psychological distress using effective measures to prevent psychological symptoms and to promote socio-emotional skills in the school context.

## 1. Introduction

Since COVID-19, the literature has increasingly sought to reflect on the health of young people and the impact of the pandemic on this population [1,2,3]. Young people report negative psychological, physical, and social effects of the pandemic [4]. In terms of psychological health, studies show that young people feel lonelier and present increased symptoms of depression, anxiety, and stress [5,6,7,8].

At a national level, both the Directorate-General for Education and Science Statistics (DGEEC) study entitled “Psychological Health and Well-being in Portuguese Schools” and the World Health Organization (WHO) study “Health Behaviour in School Aged Children (HBSC)” which extends to an international level [9], have been fundamental for the characterization of the psychological health and well-being of Portuguese adolescents before and after COVID-19 [10,11].

Studies carried out by Matos et al. [11] estimated that one-third of the adolescents reported complaints about their psychological health. Compared to 2018, in 2022, this percentage increased, with 11.6 and 21% of young people reporting symptoms of psychological distress and lower life satisfaction [11].

Several studies about mental health in children and adolescents focus mainly on psychopathological symptoms, even in non-clinical samples. However, there are other factors that can interact with psychological symptoms and distress and can contribute to characterizing at-risk and high-risk children and adolescents.

The Dual Model [12,13,14,15,16,17,18,19] has proven to be useful in understanding the psychological health of the younger population, encompassing two dimensions associated with psychological health and psychological distress. According to this model, tested in non-clinical and clinical samples, lower results on psychopathology measures are not enough to affirm that children and adolescents present positive mental health outcomes; in addition, according to this model, children and adolescents with negative mental health outcomes can score high in measures of well-being or life satisfaction.

This model, adapted by Matos et al. [12] and named in the same study as the Quadripartite Model, proved to be relevant in identifying associations that promote or impair children and adolescents’ well-being. According to this model, it is possible to define four psychological states: complete psychological health (reduced psychological symptoms and high life satisfaction), incomplete psychological health (reduced psychological symptoms and low life satisfaction), incomplete psychological distress (prominent psychological symptoms and high life satisfaction), and complete psychological distress (prominent psychological symptoms and low life satisfaction). Thus, complete psychological health implies the presence of both conditions: high life satisfaction and reduced psychological symptoms of distress [12,13,14,15,16,17,18,19].

In the previous study by Matos et al. [12], the obtained results proved the usefulness of this model to identify the distribution of young people in each of the situations defined by conditions of high or low life satisfaction and high or low presence of psychological symptoms of distress [13,14,15,16,17,18,19]. In this study, it was possible to observe significant differences between the four groups that emerged from the Quadripartite Model. The group with complete psychological health (no-risk, with higher levels of life satisfaction and reduced levels of psychological symptoms) included more male and younger students. On another axis, the group with complete psychological disorder (high risk, with higher levels of psychological symptoms and reduced levels of life satisfaction) included more young girls and students with higher levels of education.

The Quadripartite Model [12] not only highlighted gender and age differences but also emphasized a significant association between each of these four groups and various ecosystems of young people’s lives (i.e., family, school), as well as other factors linked to their health (i.e., socio-emotional skills, physical activity, sleep, screen time), and corroborated previous studies [19,20,21]. The study also highlights, among other factors, the impact of the COVID-19 pandemic and the impact of the school environment on the psychological health of this population, given that, from 2018 to 2022, psychological symptoms increased and life satisfaction decreased [12].

Based on the previously obtained results, the present study seeks to extend the understanding of the factors associated with well-being and psychological symptoms, based on the quadripartite model, which constitutes a useful tool for the identification and dissemination of recommendations for prevention/promotion measures.

Thus, the present study had the main goal of identifying the individual and contextual factors that could discriminate the students based on well-being and psychological symptom scores.

## 2. Materials and Methods

### 2.1. Participants

A total of 3073 students from lower and upper secondary education (2nd and 3rd cycles of basic education and secondary education), 49.5% female, with a mean age of 13.5 years old (SD = 2.37), attending grades 5 to 12 in school groups within all regions of the country, participated in this research (see Table 1).

Participants were geographically distributed across mainland Portugal: 35.5% from the North, 25.2% from the center, 19.4% from the Lisbon and Tagus Valley and West region, and 20.9% from the South. Complementary information about the sample can be obtained from Matos et al. [10].

### 2.2. Measures

The research protocol used in this second wave of the study was similar to the one used before and included questions related to sociodemographic data and a set of indicators of psychological health and well-being, relating to (i) life satisfaction, (ii) well-being, (iii) psychological distress, (iv) emotional skills, (v) impact of the pandemic, (vi) stress, anxiety, and depression, and (vii) positive development. Information on these indicators and the range of responses can be seen in Table 2.

Life satisfaction was assessed using the Cantril scale [22], in which participants rated their perception of their own life on a scale ranging from 0 to 10. Higher results correspond to the greatest possible life for the individual.

Well-being was assessed by the WHO-5 Well-Being Scale [23], consisting of five items answered on a 5-point Likert scale (0. Never; 4. All the time). Higher results correspond to higher well-being. The psychometric properties in the Portuguese population are available in Matos et al. [10].

The assessment of symptoms of psychological distress [24] involved the use of a 5-point Likert scale (1 = rarely or never and 5 = almost every day) consisting of five items. Higher results correspond to more symptoms. The psychometric properties in the Portuguese population are also available in Matos et al. [10].

Socio-emotional skills were assessed by the Survey on Social and Emotional Skills (SSES) [25], composed of 104 items, combined in 15 dimensions: optimism, emotional regulation, resilience/stress resistance, confidence, curiosity, sociability, persistence/perseverance, creativity, energy, cooperation, self-control, sense of belonging to school, bullying, relationship with teachers and test anxiety. Participants answered the items using a 5-point (0. Strongly disagree; 4. Strongly agree) or a 4-point Likert scale (0. Never or almost never; 3. Once a week or more). Higher results correspond to more socio-emotional skills. Adequate internal consistency values were obtained in the Portuguese version, varying from 0.68 to 0.80. The psychometric properties in the Portuguese population are available in Matos et al. [10].

Depression, anxiety, and stress were assessed using the Depression, Anxiety, and Stress Scale (DASS-21) [26,27,28], a self-report composed of 21 items combined in 3 dimensions. Participants responded to the items using a 4-point Likert scale (0 = does not apply to me at all and 3 = applies to me most of the time). Higher results correspond to higher levels of depression, anxiety, and stress. Adequate internal consistency values were obtained in the original and in the adapted version and varied from 0.84 to 0.89 and from 0.74 to 0.78, respectively [26,27,28].

Positive youth development (PYD) was assessed through four of the dimensions of the PYD scale [29,30]: competence, confidence, connection, and contribution, composed of 6, 6, 5, and 8 items, respectively. Answers to each of the items are given in a 5-point Likert scale (0 = strongly disagree and 4 = strongly agree, and 0 = never true and 4 = always true). Higher results indicate higher levels of competence, confidence, connection, and contribution. Adequate internal consistency values, Cronbach’s α, were reported in the original and in the adapted version [29,30], varying from 0.80 to 0.92 and 0.80 to 0.87, respectively.

### 2.3. Procedure

The second wave of the study of the Psychological Health and Well-being Observatory (OSPBE), coordinated by Matos and colleagues [10] and promoted by the DGEEC, was conducted in 2024. Its primary aim was to monitor indicators of psychological health and well-being in school settings two years later, to support the development and implementation of preventive and promotive measures across the educational community.

Data collection was, once again, based on a nationally representative sample involving school clusters and individual schools across mainland Portugal, randomly selected according to the NUTS III territorial classification. Participation was voluntary, and schools were invited to collaborate upon expressing their interest.

All instruments were administered digitally, ensuring anonymity and confidentiality of responses. Informed consent was obtained from all participants or from their legal guardians in cases involving minors. The study adhered to the ethical principles of research in educational contexts, including data protection, informed consent, and the safeguarding of participants’ privacy and well-being. More information about the study procedures is available online at https://info.dgeec.medu.pt/saude-psicologica-e-bem-estar-2024/ (accessed on 29 June 2025).

### 2.4. Statistical Procedures

Data were analyzed using SPSS 25.0 (SPSS, Chicago, IL, USA). Raw scores for each variable were used. Cluster analyses without previously defined clusters were performed to identify the groups. Subsequent multivariate variance analyses, using the Tukey method for multiple-group comparisons, were undertaken to confirm group differences. In each of the analyses, the basic assumptions for carrying out this type of procedure were taken into consideration (sample and group size, normality, and homogeneity of variances). Subsequently, to analyze the variables that best discriminated the groups, a multivariate discriminant analysis was also performed using the stepwise method. The discriminant functions (linear combinations of predictor variables that are used to distinguish between two or more groups) presenting larger eigenvalues were considered for the results. A confidence level of 95% was considered for each of the analyses.

## 3. Results

### 3.1. Cluster Analysis

A two-step cluster analysis was conducted based on the total score of psychological symptoms and on the total score of well-being and ran with no predefined clusters. The final cluster centers evidenced four distinct groups: Cluster 1, complete psychological health (CPH, no-risk) was characterized by high well-being (M = 20.81) and reduced psychological symptoms (M = 1.72) and Cluster 2, incomplete psychological distress (IPD, at-risk) represented participants with high well-being (M = 17.17) and prominent psychological symptoms (M = 9.94); Cluster 3, incomplete psychological health (IPH, at-risk), represented participants with low well-being (M = 14.03) and reduced psychological symptoms (M = 4.05) while Cluster 4, complete psychological distress (CPD, high-risk) was characterized by low well-being (M = 8.83) and prominent psychological symptoms (M = 12.68) (see Table 3).

**Chart 1:**
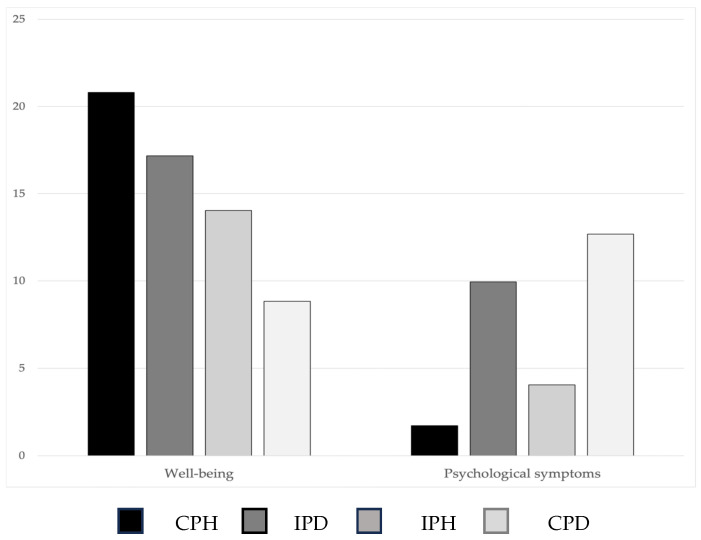
Group comparisons for well-being and psychological symptoms according to clusters. CPH = Complete Psychological Health; IPD = Incomplete Psychological Distress; IPH = Incomplete Psychological Health; CPD = Complete Psychological Distress.

Subsequent variance analyses results revealed significant group differences for both variables, F (2873, 3) = 2494.83, *p* < 0.001 (well-being), and F (2873, 3) = 2787.44, *p* < 0.001 (psychological symptoms). Multiple comparisons between the four groups showed that all groups were different, *p* < 0.001 (see Chart 1).

The final solution allocated 842 students to Cluster 1, 673 to Cluster 2, 792 to Cluster 3, and 570 to Cluster 4, excluding only 6.4% of the cases.

### 3.2. Multivariate Discriminant Analysis

Multivariate analyses of variance were first performed to study the differences between the groups obtained through cluster analysis. Comparisons between the three groups showed the existence of statistically significant differences in all studied variables, so all variables were considered in the discriminant analysis.

Following the obtained results, in the multivariate discriminant analysis, using the stepwise method, all the variables previously studied were included as independent variables.

The results of the discriminant analysis showed the existence of three statistically significant discriminant functions: the first function, Wilks’ λ = 0.41, χ^2^ (39) = 1990.51, *p* = 0.0005; the second function, Wilks’ λ = 0.91, χ^2^ (24) = 216.07, *p* = 0.0005; and the third function, Wilks’ λ = 0.97, χ^2^ (11) = 82.16, *p* = 0.0005, explaining 92.4 and 4.7% of the variance.

Although there are no fixed rules for the interpretation of eigenvalues in multivariate discriminant analysis, only the first two functions were included in the analysis, due to the low eigenvalue of the third function, and because larger eigenvalues indicate a stronger ability of that function to differentiate between groups. This decision was taken to capture a greater proportion of the variance between the groups compared to the variance within each group.

The obtained canonical correlations were equal to 0.74 and 0.24 in each discriminant function, indicating that 54.8% and 5.76% of the variance was explained by the relationship between the associated variables and group membership in function 1 and function 2, respectively. The coordinates of the centroids of the four groups in the two discriminant functions are presented in Table 4.

**Figure 1:**
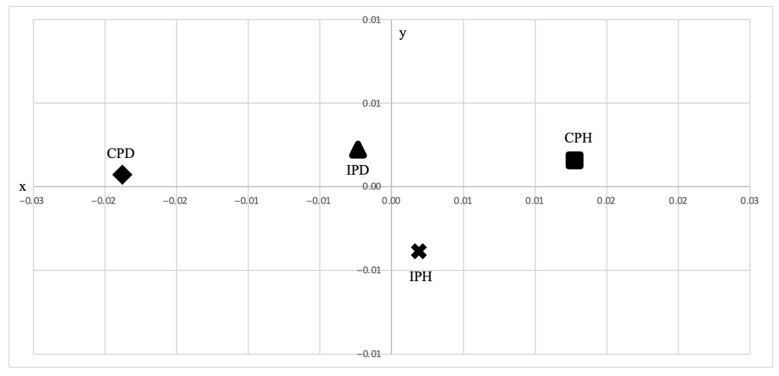
Graphical representation of group centroids.

From these coordinates, a graph was created that represents the four groups (CPH, IPD, IPH, CPD) by their centroids in the space of the discriminant functions (see Figure 1).

The graphical representation of the two functions discriminated between the four groups: students in the CPH group (no-risk) were represented on the positive side in both functions (x, y); students in the IPH (at-risk) group were represented on the positive side by function 1 (x) and on the negative side by function 2 (y); finally, students included in the CPD (high-risk) and IPD (at-risk) groups were represented on the negative side by function 1 (x) and on the positive side by function 2 (y).

The graphical representation of the two functions showed that the first function discriminated between students with CPD and IPD and students with CPH, while the second function discriminated between students with IPH and students with CPD, IPD, and CPH.

The description of each of the functions based on the variables studied, ordered by the amplitude of the correlations with the two discriminant functions, can be observed in Table 4. Correlations with a value equal to or greater than 0.30 were considered statistically significant [31].

Discriminant function 1 (x) shows higher correlations with optimism, emotional control, energy, connectedness, confidence, sense of belonging at school, and resilience. Discriminant function 2 (y) shows higher correlations with stress, depression, energy, contribution, and persistence/perseverance (see Table 4).

**Figure 2:**
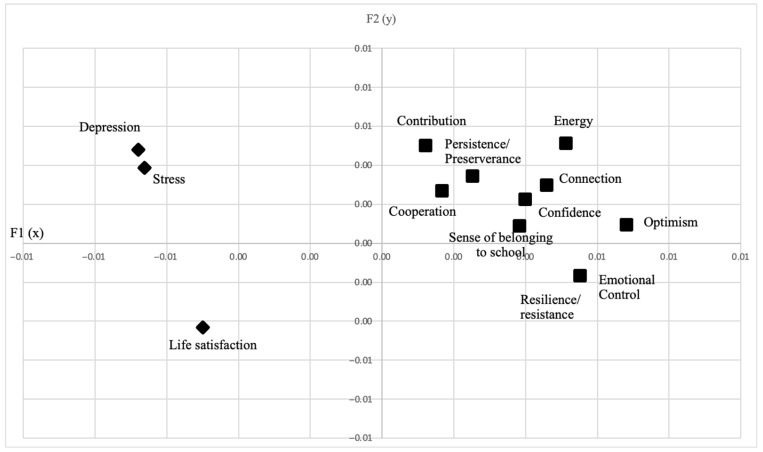
Graphical representation of correlations with discriminant functions.

Thus, the first (x) and second (y) functions were defined on the positive side by contribution, persistence/perseverance, energy, connection, confidence, sense of belonging to school, cooperation, and optimism, while the second function (y) was defined, also on the positive side, by depression. The first function (x) was also defined on the negative side by depression and stress, while the second function (y) was defined on the negative side by life satisfaction (see Figure 2).

The results showed that students in the CPH group (no-risk, represented on the positive side in both functions) were characterized by more socio-emotional skills and positive youth development, and students included in the CPD group (high-risk, represented on the negative side by function 1 and on the positive side by function 2) were characterized by higher stress and depression symptoms.

**Chart 2:**
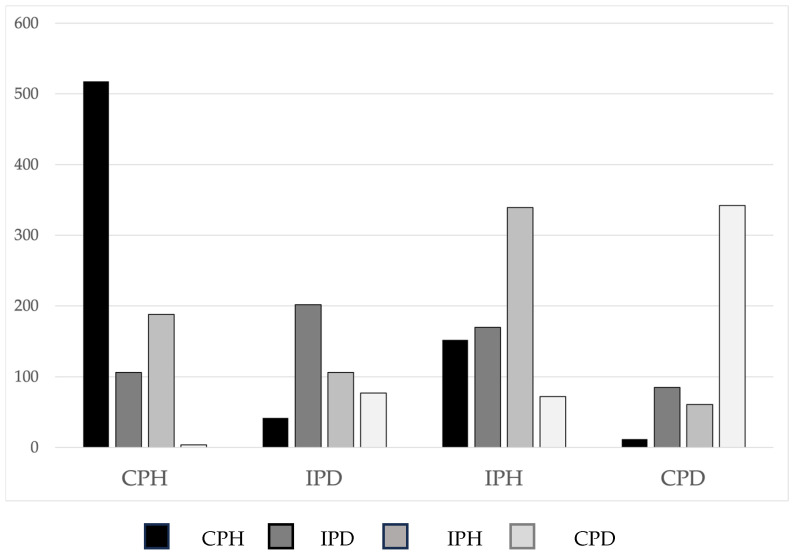
Classification results. CPH = Complete Psychological Health; IPD = Incomplete Psychological Distress; IPH = Incomplete Psychological Health; CPD = Complete Psychological Distress.

Additionally, Chart 2 shows that the discriminant function analysis indicates that 71.59% of the students in the CPH group, 35.9% of the students in the IPD group, 48.8% of the students in the IPH group, and 69.1% of the students in the CPD group were correctly classified.

The comparison of the measure of the classification power of the discriminant function (Press Q = 55.14) with a critical value based on the chi-square distribution (6.63; *p* < 0.01) showed that the percentage of correct classifications is significantly higher than what would be expected due to chance, so it is possible to interpret the discriminant functions with the aim of developing group profiles.

Thus, the overall classification results, with 56.6% of participants correctly classified, showed that students in the CPH group differed from the students in the CPD in terms of contribution, persistence/perseverance, energy, connection, confidence, sense of belonging to school, cooperation, and optimism; emotional skills were higher in the first group. In turn, students in the CPD (high-risk) group differed from the students in the CPH (no-risk) group in terms of high stress and depression and low life satisfaction (See Figure 2). However, according to these results, we could not discriminate between students in the IPD and IPH (at-risk) groups.

## 4. Discussion

The main goal of the present study was to identify the student profiles according to high and low well-being and high and low psychological symptoms.

Results of cluster analysis allowed allocation of the students to four groups without previous constraints, CPH (no-risk), IPH, IPD (at-risk), and CPD (high-risk), which were consistent with previously obtained results based on the quadripartite model [12,13,14,15,16,17,18,19,20,21] and evidenced the importance of the assessment, in a continuum, of psychological symptoms simultaneously with well-being, in order to capture main features of mental health.

Over the years, mental health problems have been assessed, in most cases categorically, through indicators of psychological problems. The quadripartite model, a continuous approach that considers other relevant factors for mental health, specifically life satisfaction or well-being (e.g., [13,14,15]), overcomes this gap, assessing two distinct dimensions of psychological health, which can help researchers and practitioners in the early identification of at-risk students with either low or high life satisfaction paired with either low or high psychological problems. Thus, the quadripartite or dual model can be a relevant tool for substantiating differences between no-risk, at-risk, and high-risk students.

The obtained profiles for each group were also in agreement with previous literature (e.g., [1,12,16,17,20,21,32]). However, also according to previous literature (e.g., [33,34,35]), results evidenced a better discrimination of the extreme groups, no-risk and high-risk, with no-risk students evidencing more emotional skills and positive development, and high-risk students evidencing higher psychopathology and low life satisfaction. These results point to the need for replication of this model with the aim of discriminating the profiles of each of the four groups, including other relevant variables, which may contribute to distinguishing at-risk students from those of the other groups.

At-risk students, whether IPH or IPD, may present a combination of common and different risk factors [36], and the stability of both profiles is usually lower, e.g., [33,34,35]. In a systematic review of the literature on the scientific evidence for using the dual model [36], results demonstrated psychometric substantiation of the two-dimensionality of the dual-factor model, which was shown to classify most of the two extreme groups over time. Thus, it is possible that, in the intermediate groups, socio-emotional development is interfaced with other contextual (e.g., family communication, neighborhood safety), demographic (e.g., age, gender), and individual factors (e.g., attachment, resilience, chronic disease), which were not assessed in the present study. These factors interact with each other, resulting in positive or negative outcomes, and should be considered in future studies to improve the impact of preventive programs.

Despite the importance of the results obtained, the present study has some limitations related to the use of self-report measures and its cross-sectional nature, which does not allow the interpretation of the results obtained in terms of their cause–effect relationship.

However, once again, results point to the need to assess risk, vulnerability, and protection factors, as well as the stability and consequences of IPD and CPD when promoting mental health in students. Thus, public policies regarding students’ health should include, in the formal and non-formal school curriculum, (i) universal interventions, targeted to promote socio-emotional competences for all students (no-risk, at-risk, and high-risk) regardless of any risk factor; (ii) selective interventions, targeted to prevent unwellness and lower mental distress in at-risk students; and (iii) indicated programs, targeted at preventing high-risk students from developing further symptoms as well as reducing CPD severity when there are already early signs. Also, public policies should consider teacher training in socio-emotional skills as well as socio-emotional skills training for school staff (teachers and non-teachers) and the promotion of healthy work environments. The adoption of an intergenerational approach, involving students, families, and community participation, can contribute to an integration of the different ecosystems involved in the students’ lives. Only public policies that take all these factors into account and systematically develop, implement, and assess these actions based on a comprehensive and steeped care framework, in the school context in articulation with other contexts (e.g., health, justice, social service), will present efficacy in reducing or preventing psychological health problems and promoting psychological health in children and adolescents.

## Figures and Tables

**Table 1:** Demographic characteristics of the sample.

	Number of Participants	%	Mean	Standard Deviation
Gender				
Male	1489	48.5		
Female	1522	49.5		
Other	25	0.8		
Prefer not to answer	37	1.2		
School grade				
5th grade	357	11.6		
6th grade	418	13.6		
7th grade	420	13.7		
8th grade	391	12.7		
9th grade	442	14.4		
10th grade	326	10.6		
11th grade	394	12.8		
12th grade	325	10.6		
Age	2996	97.5	13.49	2.37

**Table 2:** Measures and variables under study (adapted from Matos et al. [2]).

Variables	Category/Number of Items	Min and Max
Gender	Boy; Girl; Other; Prefer not to answer	
Age	-	9 to 18 years old
Grade	From 5th to 12th	5th to 12th grade
Life satisfaction	11-step ladder	0 = worst possible to 10 = best possible life satisfaction
Well-being	5 items	5 (lower) to 15 (higher well-being)
Psychological symptoms	5 items	5 (lower) to 15 (higher psychological symptoms)
**Socio-Emotional Skills**		
Optimism	8 items	0 = Strongly disagree to 4 = Strongly agree 0 (lower) to 32 (higher socio-emotional skills)
Emotional regulation	8 items	
Resilience/stress resistance	8 items	
Confidence	8 items	
Curiosity	8 items	
Sociability	8 items	
Persistence/Perseverance	8 items	
Creativity	8 items	
Energy	8 items	
Cooperation	8 items	
Self-control	8 items	
Sense of belonging to school	6 items	
Bullying	4 items	0 = Never or almost never to 3 = Once a week or more 0 lower to 12 higher bullying 0 worst to 9 better relationships with teachers
Relationship with teachers	3 items	
Test anxiety	3 items	0 = Strongly disagree to 4 = Strongly agree 0 (lower) to 12 (higher test anxiety)
Stress	7 items	0 = Did not apply to me at all to 3 = Applied to me most of the time 0 (lower) to 21 (higher stress, anxiety and depression)
Depression	7 items	
Anxiety	7 items	
Competence	6 items	(used for most items): 0 = Strongly disagree to 4 = Strongly agree (used only for items i and j): 0 = Never true to 4 = Always true
Confidence	6 items	
Connection	8 items	
Contribution	5 items	0 = No time to 4 = 6 or more hours 0 (lower) to 24/32/20 (higher competences)

**Table 3:** Cluster profiles and centroids.

Cluster 1	Cluster 2
Complete Psychological Health	Incomplete Psychological Distress
(N = 842; 29.3%)	(N = 673; 23.4%)
High well-being (M = 20.81)	High well-being (M = 17.17)
Reduced psychological symptoms (M = 1.72)	Prominent psychological symptoms (M = 9.94)
Cluster 3	Cluster 4
Incomplete Psychological Health	Complete Psychological Distress
(N = 792; 27.5%)	(N = 570; 19.8%)
Low well-being (M = 14.03)	Low well-being (M = 8.83)
Reduced psychological symptoms (M = 4.05)	Prominent psychological symptoms (M = 12.68)

Note: N = Number of participants; M = Mean.

**Table 4:** Coordinates of the centroids of the groups for the two discriminant functions and variables ordered by the amplitude of the correlations with the discriminant functions.

Group	Function 1	Function 2
CPH	1.28	0.16
IPD	−0.23	0.22
IPH	0.19	−0.39
CPD	−1.88	0.07
Variables	Function 1	Function 2
Optimism	0.68	0.09
Stress	−0.68	0.48
Depression	−0.66	0.39
Emotional control	0.56	−0.16
Energy	0.51	0.51
Life satisfaction	−0.50	−0.43
Connection	0.46	0.29
Confidence (SSES)	0.40	0.22
Sense of belonging to school	0.38	0.09
Contribution	0.12	0.50
Persistence/perseverance	0.25	0.34
Cooperation	0.17	0.26

CPH = Complete Psychological Health; IPD = Incomplete Psychological Distress; IPH = Incomplete Psychological Health; CPD = Complete Psychological Distress.

## Data Availability

The data presented in this study are available on request from the corresponding author. The data are not publicly available due to privacy and ethical reasons.

## Abbreviations

The following abbreviations are used in this manuscript:

CPD	Complete Psychological Distress.
CPH	Complete Psychological Health
DGEEC	Directorate-General for Education and Science Statistics
HBSC	Health Behavior in School-Aged Children
IPD	Incomplete Psychological Distress
IPH	Incomplete Psychological Health
OECD	Organization for Economic Co-operation and Development
OSPBE	Psychological Health and Well-being Observatory
WHO	World Health Organization
